# The use of air-lift adsorber with a floating filling from a cross-linked chitosan hydrogels for Reactive Black 5 removal

**DOI:** 10.1038/s41598-021-92856-y

**Published:** 2021-06-28

**Authors:** Tomasz Jóźwiak, Urszula Filipkowska

**Affiliations:** Department of Environmental Engineering, University of Warmia and Mazury in Olsztyn, Warszawska St. 117a, 10-957 Olsztyn, Poland

**Keywords:** Environmental chemistry, Chemical engineering, Polymer chemistry

## Abstract

This work substantially extends knowledge on the possibilities of treating colored industrial wastewater via sorption under flow conditions. The presented study aimed to determine the effectiveness of Reactive Black 5 (RB5) dye sorption from aqueous solutions under dynamic (flow) conditions in an unconventional air-lift type loop reactor with a filling made of hydrogel chitosan sorbents. The dye was removed from mono-component solutions (deionized water + RB5) and synthetic dyeing wastewater containing RB5 dye, NaCl (3 g/L), and an anti-creasing agent—UNICREASE JET (2 g/L). The sorbents tested in the study included: unmodified chitosan (CHs), chitosan ionically cross-linked with sodium citrate (CHs-CIT), and chitosan covalently cross-linked with epichlorohydrin (CHs-ECH). Each experimental series aimed to determine: the bed break-through time (C_E_ = 0.1 C_0_), time of depletion of the sorbent’s sorption properties (C_E_ = C_0_), and maximal sorption capacity of the sorbents (Q_max_). The data obtained under dynamic conditions were described using Thomas, Yoon–Nelson, and Bohart–Adams models. The volume of the solution effectively treated in the air-lift reactor was significantly affected by chitosan sorbent type. At C_0_ = 50 mg RB5/L, the adsorber with the filling made of 1 g d.m. CHs allowed for the effective treatment of 4.6 L of synthetic wastewater (Q_max_ = 1504.7 mg/g), whereas CHs-ECH ensured 34.6 L of the treated solution (Q_max_ = 3212.9 mg/g).

## Introduction

Several industrial activities, such as textile, tanning, and paper industries, generate the colored wastewater. As this wastewater may contain sparingly-biodegradable dyes, it is usually treated using physicochemical technologies, such as electrocoagulation, electrochemical oxidation or photodegradation^1–4^. Many scientists share a common opinion that sorption processes are the most environmentally-friendly methods for wastewater decolorization^5^. Activated carbons are the most frequently used sorbents because of their high sorption capacity owed to, i.a., very large surface, often exceeding 2000 m^2^/g^6^. Other efficient sorbents often used in the industry include ion-exchange resins that feature high stability and regenerability^7^. Drawbacks of the mentioned sorbents, however, include their relatively high price and the impossibility of using them in adsorbents with a floating filling.

Chitosan-based sorbents represent a very good alternative to the commercial activated carbon. Chitosan is a polysaccharide produced from chitin, which is the most ubiquitous biopolymer in the natural environment right after cellulose^8^. Once properly prepared, it can exhibit a multiply higher sorption capacity than the commercial activated carbons^9^. With regard to anionic dyes, which are the most common contaminants found in wastewater from the dyeing industry, the sorption capacity of chitosan-based sorbents can reach up to Q_max_ > 2000 mg/g^10^, mainly due to their basic character, being unusual for biopolymers, caused by the presence of amine groups in their structure. The positively-charged surface of chitosan sorbents interacts electrostatically with dye anions, thereby significantly enhancing dye sorption^11^.

Chitosan sorbents can take the form of a powder, flakes, or hydrogel beads, with the latter showing the best sorption properties. The polysaccharide chains of the hydrogel are spaced apart, and the spaces between them are filled with water, which allows the sorbates to penetrate into the sorbent and use sorption centers more effectively. Compared to chitosan in the form of flakes and powder, the sorption of contaminants on chitosan hydrogel beads is largely due to absorption^12^. The advantage of chitosan in this form is the convenience of its application and removal from the solution after the treatment process. This is especially useful in adsorbers with a floating filling. The hydrogel form of chitosan additionally enables easy modification of the sorbent, e.g. cross-linking.

Chitosan hydrogels can be used for the sorption process in both static and dynamic (flow) conditions. Their sorption capacity may vary significantly depending on the conditions in the system. Findings available in the literature on the sorption of dyes on chitosan sorbents derive mainly from experiments performed under static conditions, while respective investigations under dynamic conditions are still few, even though they much better simulate the processes of wastewater decolorization in industrial plants.

The cross-linking of chitosan ensures its stability in a strongly acidic environment and may also improve its sorption properties^13^. It can be covalent or ionic, depending on the cross-linking agent used. The covalent cross-linking of chitosan is a chemical reaction resulting in the formation of permanent covalent bonds between the cross-linking agent and the polysaccharide chains^14^. One of the best covalent cross-linkers is epichlorohydrin. A properly carried out process of chitosan cross-linking with epichlorohydrin ensures the complete stability of the hydrogel even at very low pH values (pH < 2) and boosts its sorption properties. The disadvantages of this type of cross-linking include greater hardness and brittleness of the sorbent, which increases the risk of its mechanical damage in systems with a floating filling^15^. During ionic cross-linking, electrovalent (ionic) bonds are formed between the cross-linking agent and the chitosan chains. In the aqueous solution, the ionic cross-linking agent has a charge opposite to that of the modified polymer. The electrostatic attraction of the polymer chains to the ionic cross-linking agent causes the ionic cross-linking effect^16^. One of the most commonly used ionic cross-linkers is sodium citrate^17^. The ionically-linked polysaccharide chains of chitosan are responsible for the compact structure of the sorbent. Compared to the covalently cross-linked chitosan, the ionically cross-linked one is less stable in acidic solutions (pH 2–3). The structure of the ionically cross-linked chitosan hydrogel is, however, more flexible and resistant to mechanical damage^15^.

The most frequently used type of reactors in the flow systems are column adsorbers (beds with an immobilized filling)^18–20^. They usually consist of a wide pipe/tube filled with an adsorbent, through which the liquid to be treated flows. Unfortunately, they have some drawbacks, like the high risk of the appearance of areas with increased or reduced liquid flow, leading to ineffective use of the sorption material, and the potential risk of sorbent damage by gravitational forces and the flow of an aqueous solution^21^.

An air-lift reactor can represent an alternative to the column adsorber. Unlike the latter, it is a type of a bed with a moving filling. These reactors are often in the shape of a tube with inlet ports mounted at the bottom to supply the treated liquid and gas (air). The gas flowing into the system forces the sorbent to move^22^. It is assumed that the air-lift adsorber efficiently uses the sorbent due to the similar dye concentration in the entire reactor’s volume and that the risk of sorbent structure damage is small.

Most of the research conducted so far on dye sorption concerned the removal of dye from "mono-component" aqueous solutions (distilled water + dye). The results of such research may, however, give a false view of the sorption properties of the tested sorbents in the case of real industrial wastewater, which may contain substances (salts, hydrocarbons) that impair dye binding to sorbents. Therefore, it seems advisable to investigate the decolorization process of aqueous solutions, whose composition resembles that of the authentic industrial wastewater.

This study aimed to determine the effectiveness of a common industrial dye—Reactive Black 5—sorption from synthetic wastewater and from solutions based on distilled water under flow conditions in the air-lift adsorber with the filling made of chitosan gel, using three types of the hydrogel filling: unmodified chitosan, chitosan covalently cross-linked with epichlorohydrin, and chitosan ionically cross-linked with sodium citrate.

## Materials and methods

### Materials and equipment

#### Chitosan

The sorbents used in the experiments were prepared from flakes (1–2 mm) of chitosan sourced from shrimp exoskeletons (Heppe Medical Chitosan GmbH, Halle, Germany). As declared by the producer, the chitosan deacetylation degree was DD = 82.6–87.5% (DD = 85.0% on average), the viscosity of 1% chitosan in 5% acetic acid was 500 mPas, and the total content of heavy metals (Pb, Hg, Cd) was at < 25 ppm.

#### Reactive Black 5 dye

Reactive Black 5 (RB5) was purchased from the Dye Producing Plant “Boruta” SA (Zgierz, Poland). Its characteristics provided by the producer is presented in Table 1.

**Table 1:** Characteristics of RB5.

Structural formula	Chemical formula	C_26_H_21_N_5_Na_4_O_19_S_6_
	Molecular weight	991.8 g/mol
	Dye type	acidic (anionic–reactive)
	Dye class	diazo
	Characteristic functional groups	vinylsulfone
	**λ**_**max**_ (wavelength used for spectrophotometric measurement)	600 [nm]
	Uses	Dyeing of: cotton, viscose, wool, polyamide fibers
	Other commercial names	Begazol Black B, Celmazol Black B, Diamira Black B, Levafix Black E-B, Primazin Black BN, Remazol Black B

#### Chemical reagents

The following chemical reagents were used in the study:

- 99% epichlorohydrin 99% (ACROS ORGANICS, Poland)—covalent cross-linking of chitosan,
- sodium citrate dihydrate (POCH S.A., Poland)—ionic cross-linking of chitosan,
- 35–38% hydrochloric acid (POCH S.A., Poland)—solution pH correction,
- sodium hydroxide microgranules (POCH S.A., Poland)—hydrogel gelling (sorbent preparation), solution pH correction,
- analytically pure sodium chloride (POCH S.A., Poland)—preparation of synthetic wastewater,
- 99.5–99.9% acetic acid (POCH S.A., Poland)—chitosan solubilization (sorbent preparation),
- UNICREASE JET anti-creaser (TEXCHEM, Poland)—a fluid based on polyacrylamide (2%)—preparation of synthetic wastewater.

#### Laboratory equipment

The following equipment was used in the study (Table 2).

**Table 2:** Laboratory equipment used in the study.

Equipment	Function
HI 110 pH-meter (HANNA Instruments, Poland)	Solution pH correction
Water bath shaker type 357 (Elpin-Plus, Poland)	Chitosan cross-linking with epichlorohydrin
UV-3100 PC spectrophotometer (VWR spectrophotometers, Canada)	Determination of dye concentration in the solution
Peristaltic pump Minipuls 3 (MP3) (Gilson, France)	Solution feeding
Glass Rotameter Type 2000, AEA Technique, Poland	Air flow control
Air-lift reactor (adsorber)	Constructed by the research group for the need of this study, its description is provided in point 2.4

### Preparation of sorbents

25 g d.m. of chitosan flakes were dissolved in 975 g of a 5% CH3COOH solution. The resulting solution was instilled to 2M NaOH with a syringe needle (0.8 × 40) to form hydrogel beads (gelling). After 24 h, the beads were drained and rinsed with distilled water in the drain until neutral pH of the filtrate. The prepared chitosan hydrogel beads (CHs) were stored in deionized water in a laboratory cooler (4 °C).

The cross-linking of chitosan beads involved their 24-h bath in appropriately prepared solutions of cross-linking agents. During the cross-linking, a minimal dose of the cross-linking agent was used that ensured stability of the ionically cross-linked hydrogel at pH 3 and stability of the chemically cross-linked hydrogel at pH < 2 (static conditions)^10^. The dose of the cross-linking agent was 0.483 g/g_CHs_ for the chitosan hydrogel cross-linked with sodium citrate (CHs-CIT) and 0.032 g/g_CHs_ for the chitosan hydrogel cross-linked with epichlorohydrin (CHs-ECH). The cross-linking was performed in conical flasks protected with a parafilm, placed on a shaker with a water bath (150 rpm) at 22 °C for CHs-CIT and 60 °C for CHs-ECH. Similarly to CHs, the prepared CHs-CIT and CHs-ECH were stored in deionized water (4 °C). A more detailed description of the sorbent preparation procedure and complete sorbent characteristics (e.g., FTIR or dry matter of a hydrogel) have been provided in our previous works^10,15^.

### RB5 solutions

Analyses were carried out for two types of an RB5 solution. The first one (S1) was a “mono-component” solution (deionized water + RB5) with a dye concentration of 50 mg/L. The second solution (S2) was the synthetic wastewater, which contained the dye (50 mg/L) and also dyeing-aiding substances, i.e., anti-creasing agent (UNICREASE JET)—2.0 g/L, and sodium chloride—3.0 g/L. The S2 was expected to simulate post-production industrial wastewater (from the textile industry) generated immediately after the dyeing process.

The pH value of both solutions was corrected using HCl to the optimal pH of RB5 sorption, established individually for each tested sorbent in the preliminary study. Under static conditions, the optimal pH of RB5 sorption was: pH 4, pH 3, and pH 3 for CHs, CHs-CIT, and CHs-ECH, respectively^10,15^. Under dynamic (flow) conditions, pH 4 for CHs and pH 3 for CHs-CIT turned out to be too low because the swelling and partial damage of the hydrogels were observed after few days of the process. In the case of CHs-ECH, there were no signs of damage at pH 3. Ultimately, the most beneficial pH values for RB5 sorption onto CHs, CHs-CIT, and CHs-ECH under dynamic (flow) conditions were pH 5, pH 4, and pH 3, respectively.

RB5 dye concentration in the solution was measured with the spectrophotometric method using a UV–VIS 3100 PC spectrophotometer and a 10 mm JGS1 quartz cuvette, at the wavelength of 600 [nm].

### Operation of the system with the air-lift adsorber

**Figure 1:**
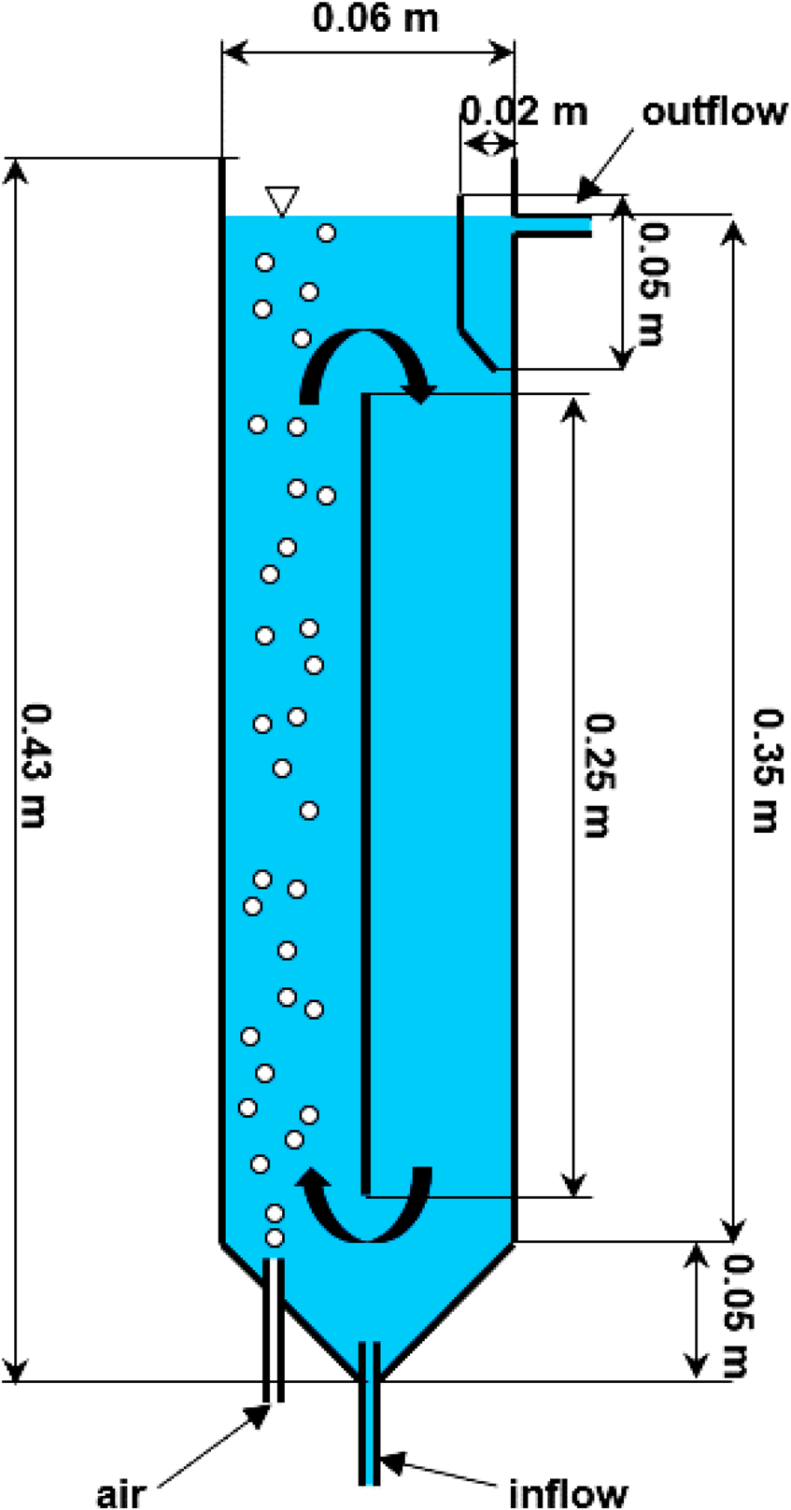
Scheme of the air-lift adsorber.

Analyses of dye sorption effectiveness under flow conditions were conducted in an air-lift type reactor having the volume of 1 L and made of plexiglass. The reactor had a circular cross-section (Φ = 0.06 m) and was 0.43 m high. A 0.25 m long partition was mounted inside the reactor. The bottom part of the reactor was in the shape of a truncated cone. Two inlet valves mounted therein served to supply air and dye solutions. The air supplied ensured the circulating motion of the solution and the sorbent around the partition. In turn, a pocket tank with an outflow valve was mounted in the upper part of the reactor. The scheme of the reactor is presented in Fig. 1.

The air was supplied to the reactors with an aerating pump generating 0.15 MPa pressure, and its flow rate in the reactor was at 50 dm3/h. This flow rate enabled sorbent mixing in the entire reactor’s volume and prevented sedimentation at the reactor’s bottom. Dye solutions were fed to the reactors using peristaltic pumps at a flow rate of 0.1 V/h (with V denoting reactor’s volume), i.e., at 0.1 L/h. The sorbent dose in the adsorber reached 1 g/L, except for the control series in which the adsorber had no filling made of the sorbent.

**Figure 2:**
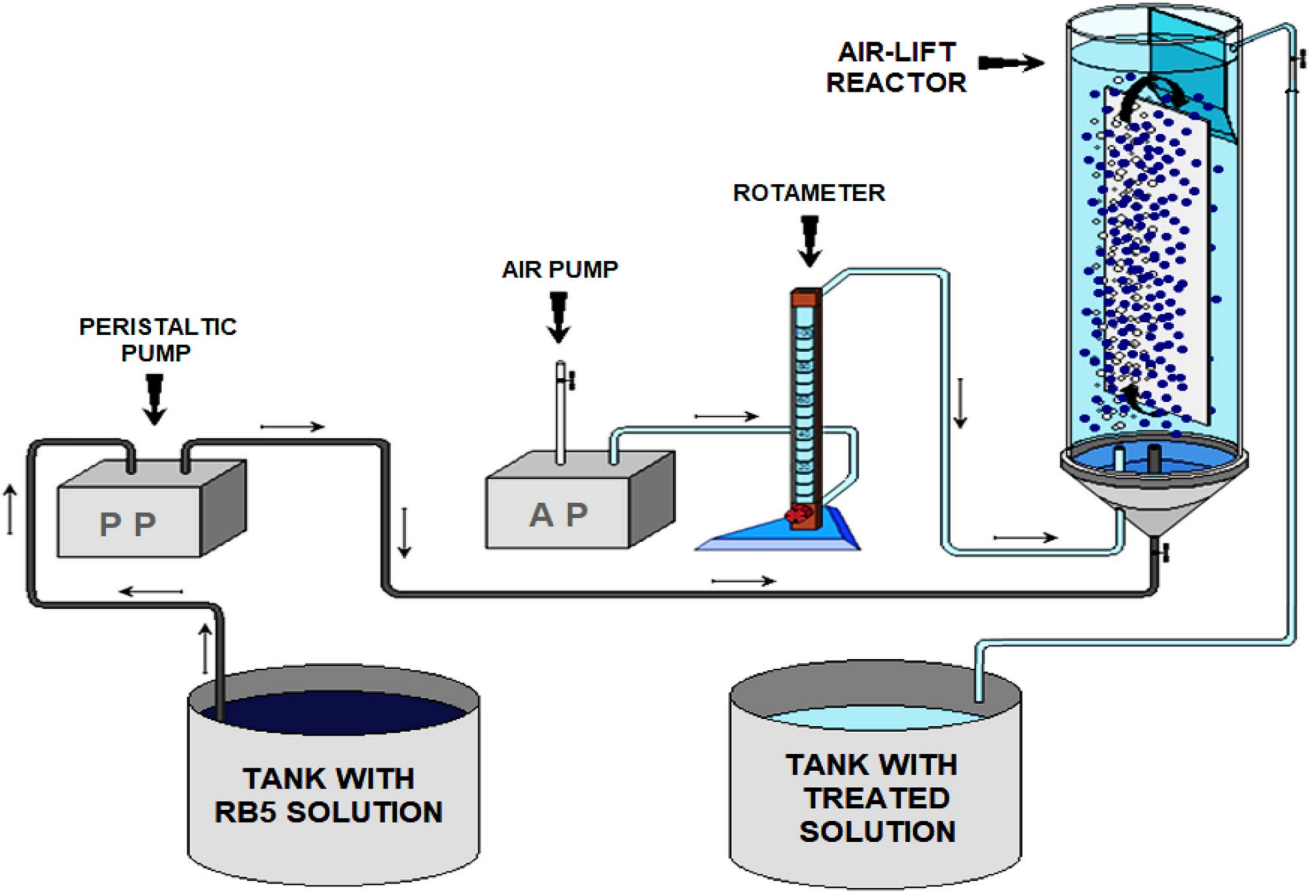
Scheme of the system with the air-lift adsorber.

A solution sample (10 mL) was collected from the pocket tank every 2 h for the spectrophotometric determination of RB5 concentration. Because the samples were clear, their centrifugation was unnecessary before their spectrophotometric analysis. The system operated in a dark room to eliminate the risk of dye photolysis in the solution. The operation scheme of the system with the air-lift adsorber is presented in Fig. 2.

### Computation methods

**Figure 3:**
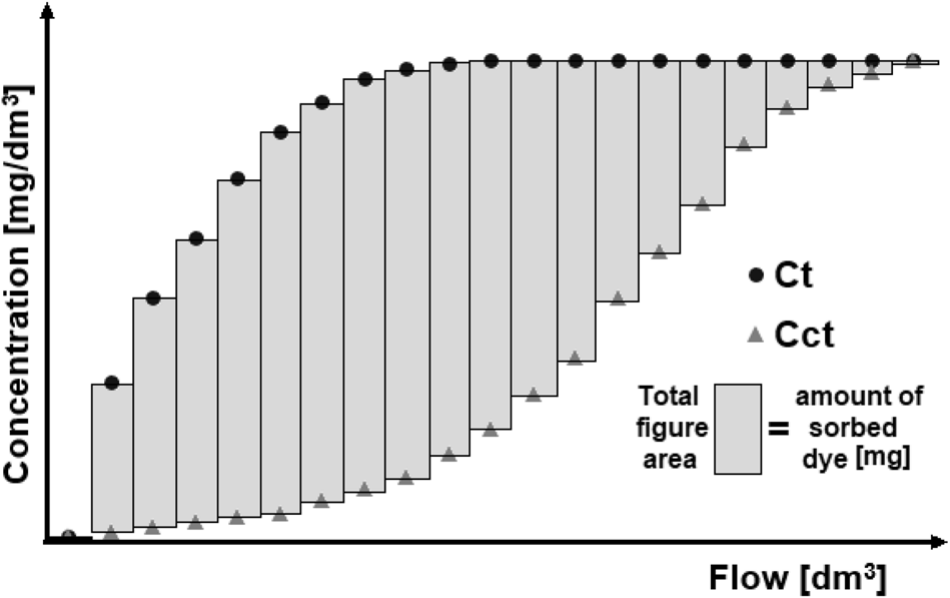
Computation scheme of the amount of dye sorbed on the sorbent over sorption time (experimental capacity).

The amount of RB5 sorbed on the sorbent under flow conditions was computed from the following formula (1). See also Figure 3.

1 $$Q=\sum_{(t=1)}\frac{\left[\left({C}_{ct}-{C}_{t}\right)\cdot Fl\cdot t\right]}{{V}_{R}\cdot {C}_{S}}$$

where Q—amount of RB5 sorbed on the sorbent after time t [mg/g d.m.], C_t_—mean RB5 concentration at the reactor’s outflow in time t (in the control sample) [mg/L], C_ct_—mean RB5 concentration at the reactor’s outflow in time t (in the samples with the chitosan sorbent) [mg/L], V_R_—reactor volume [L], Fl—RB5 solution flow rate at the reactor’s inlet [L/h], t—sorption time [h], C_S_—sorbent concentration in the reactor [g d m/L].

Three models were used to describe the results obtained under dynamic conditions: Thomas, Yoon–Nelson, and Bohart-Adams. The Thomas model is commonly used to describe adsorption dynamics in the systems based on reactors with immobilized filling^23^. It is based on the non-linear Langmuir isotherm and kinetics of the pseudo-second order reaction, and it is described with the following Eq. (2).

2 $$\frac{{C}_{t}}{{C}_{0}}=\frac{1}{1+\mathrm{e}\mathrm{x}\mathrm{p}({K}_{TH}\cdot Q\cdot \frac{X}{V}-{K}_{TH}\cdot {C}_{0}\cdot t)}$$

where C_t_—RB5 concentration at the reactor’s outlet in time t [mg/mL], C_0_—initial concentration [mg/mL], K_TH_—constant in Thomas’s model [mL/(min*mg)], Q—equilibrium amount of sorbed RB5 [mg/g], V—flow rate [mL/min], X—sorbent amount in the adsorber [g], t—time [min].

The Yoon-Nelson model assumes that the likelihood of the sorption and desorption of each sorbate molecule is proportional^24^. Computations made using this model do not require detailed data regarding the adsorbate and the adsorbent. The Yoon-Nelson model is described with the following Eq. (3).

3 $$\frac{{C}_{t}}{{C}_{0}}=\frac{1}{1+\mathrm{e}\mathrm{x}\mathrm{p}[{{K}_{YN}}^{\left(\tau -t\right)}]}$$

where C_t_—RB5 concentration at the reactor’s outlet in time t [mg/mL], C_0_—initial concentration [mg/mL], K_YN_—constant in the Yoon-Nelson’s model [1/min], t—time [min], τ—time needed to reach the half of adsorbent saturation [min].

Likewise the Thomas’s model, the Bohart-Adams model (Eq. 4) is intended for a column bed with an immobilized filling. It assumes a linear correlation between bed’s height and reactor’s working time^25^.

4 $$\frac{{C}_{t}}{{C}_{0}}=\frac{1}{1+\mathrm{exp}\left[{K}_{AB}\cdot q\cdot \frac{H}{{\vartheta }}\right]-{K}_{AB}\cdot {C}_{0}\cdot t}$$

where C_t_—RB5 concentration at the reactor’s outlet in time t [mg/mL], *C*_o_- initial concentration [mg/mL], t—time [min], q—concentration of sorbate saturation [mg/L], H—column height [cm], ϑ—flow rate [cm/min], K_AB_—kinetic rate constant in the Adams-Bohart’s model [L/(mg min)].

## Results and discussion

The experimental data obtained for RB5 sorption from S1 and S2 solutions onto CHs, CHs-CIT, and CHs-ECH under flow conditions in the air-lift type adsorber were described using three popular sorption models: Thomas, Yoon–Nelson, and Bohart–Adams. In each experiment series, the Thomas and Bohart-Adams models better described RB5 sorption than the Yoon-Nelson model, regardless of sorbent and solution tested.

**Figure 4:**
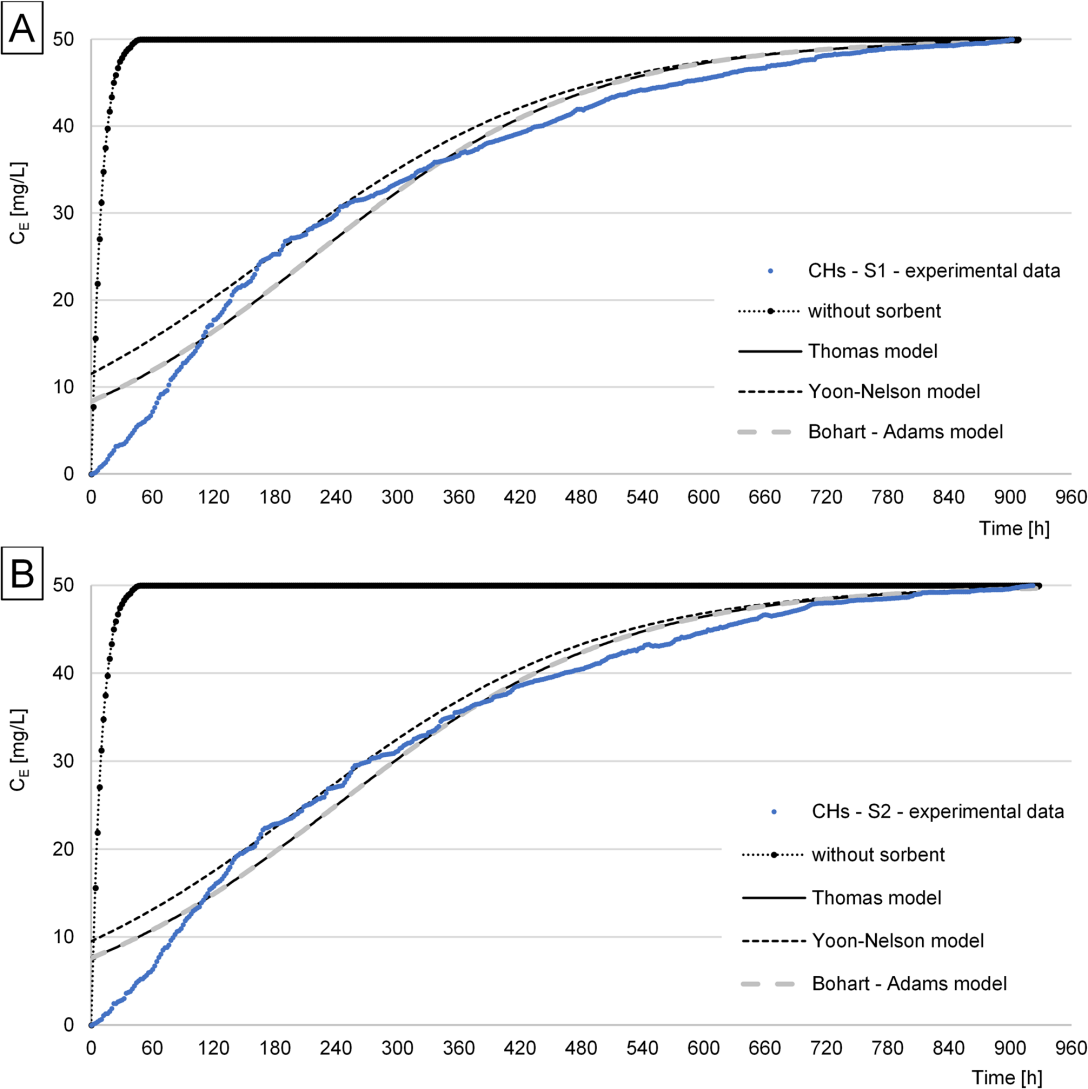
Concentration of RB5 at the outlet from the air-lift with a filling from CHs: (**A**) S1, (**B**) S2.

The effectiveness of RB5 sorption from solutions S1 and S2 in the air-lift adsorber with the filling made of CHs decreased rapidly since the beginning of reactor’s work. The experimental series performed with CHs lacked the period of a high and stable sorption effectiveness (Fig. 4). The bed break-through time (i.e. the time after which C_E_ > 0.1 C_0_) in the system with CHs was similar for both solutions tested and reached 44 h for S1 and 46 h for S2, whereas the amount of the treated solution (C_E_ < 0.1C_0_) reached 4.4 and 4.6 L, respectively. Comparable was also the time after which the sorption properties of the sorbent were depleted (i.e., the time after which C_E_ = C_0_). In the case of RB5 sorption onto CHs, it was 904 h when the dye was removed from solution S1 and 924 h when it was removed from solution S2 (Table 4). The presence of NaCl (3 g/L) and the anti-creaser (2 g/L) in S2 had a little impact on RB5 sorption onto CHs. The RB5 sorption capacity of CHs determined from the Thomas model reached 1085.9 and 1202.0 mg/g when the dye was removed from solutions S1 and S2, respectively, whereas the experimental capacity (computed from Eq. (1)—point 2.5.) was at 1390.2 and 1504.9 mg/g, respectively (Table 3). According to our previously published study^10^, the maximum RB5 sorption capacity of CHs under static conditions reached Q = 2307.0 mg/g (Table 3).

**Table 3:** Constants determined from Thomas, Yoon–Nelson, and Bohart-Adams sorption models.

Sorbent	Solution	Thomas model			Yoon-Nelson model			Bohart-Adams model			Q (exp) [mg/g]
		K_TH_*	Q [mg/g]	R^2^	K_YN_ [1/min]	τ [min]	R^2^	K_BA_*	q [mg/mL]	R^2^	
CHs	S1	0.0025	1085.9	0.968	0.00011	10,560	0.942	0.0025	1.080	0.968	1390.2
	S2	0.0024	1202.0	0.977	0.00012	12,600	0.963	0.0024	1.197	0.977	1504.7
CHs-CIT	S1	0.0027	1599.2	0.973	0.00014	16,560	0.948	0.0027	1.593	0.973	1920.9
	S2	0.0028	994.3	0.913	0.00012	8640	0.860	0.0028	0.990	0.913	1339.7
CHs-ECH	S1	0.0030	2777.3	0.994	0.00015	32,760	0.993	0.0030	2.761	0.994	3169.2
	S2	0.0030	2855.3	0.995	0.00015	34,200	0.994	0.0030	2.839	0.995	3212.9

*Unit [mL/(min mg)].

The relatively low sorption capacity of CHs noted under dynamic conditions, compared to the static ones, could be due to the higher solution pH.. The affinity of RB5 to CHs at pH 5.0 (i.e., pH used in the air-lift system) was substantially lower than at pH 4.0 (i.e., pH used under static conditions)^10^. This explains also the lack of a period of the high RB5 sorption effectiveness in the system (Fig. 4).

**Figure 5:**
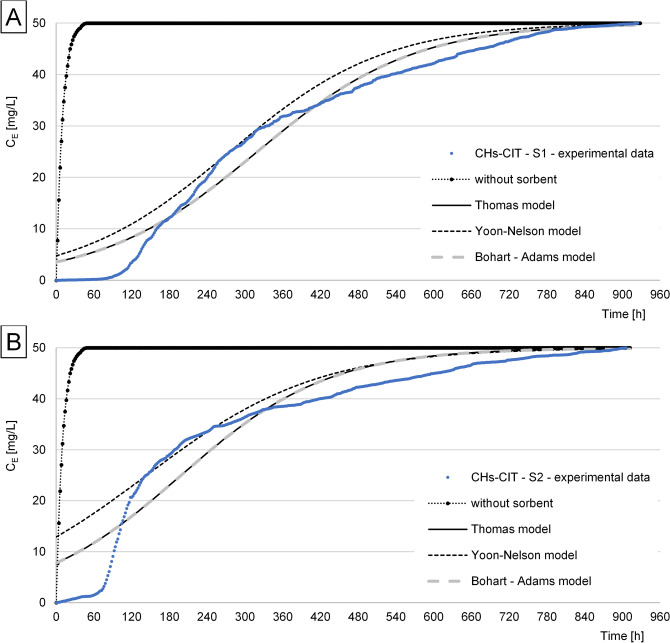
Concentration of RB5 at the outlet from the air-lift with a filling from CHs-CIT: (**A**) S1, (**B**) S2.

In contrast to the experimental series with CHs, the period of high and relatively stable RB5 sorption effectiveness could be observed in the series with CHs-CIT (Fig. 5). The bed break-through time (C_E_ > 0.1 C_0_) in the adsorber with CHs-CIT was 132 h for S1 and 82 h for S2. Like in the series with CHs, the time of CHs-CIT sorption properties depletion (C_E_ = C_0_) reached 920 h and 906 h for S1 and S2, respectively.

The composition of S2 solution had a strongt effect on RB5 sorption onto CHs-CIT. The RB5 sorption capacity of CHs-CIT computed with the Thomas model in the series with S2 reached 994.3 mg/g and was by 38% lower than in the series with S1 (Q = 1599.2 mg/g). The differences in the effectiveness of RB5 sorption onto CHs-CIT are presumably due to the anti-creasing agent, being a component of S2 solution. It contains a polyacrylamide, i.e., a polymer rich in amine groups. Presumably, it entered into reaction with citrate ions located between the chains of CHs-CIT chitosan during sorption. The anionic cross-linking agent attracted electrostatically the amine groups of the polyacrylamide. The polymer attached to the surface could impair RB5 sorption. A similar effect was not observed onto CHs nor onto CHs-ECH because of the electrostatic repulsion observed between protonated amine groups of chitosan and amine groups of the polyacrylamide. The RB5 sorption capacity of CHs-CIT determined under dynamic conditions was lower than that determined under static conditions (Table 4). Like in the case of CHs, the result obtained could probably be due to the higher sorption pH (pH 4 instead of pH 3). The weaker electrostatic interactions between RB5 and CHs-CIT at the higher pH were reflected in the lower dye sorption effectiveness^15^.

**Table 4:** Work parameters of a system with the air-lift reactor depending on the chitosan sorbent used and treated solution type.

Sorbent	Solution	Bed break-through time (C_E_ = 0.1C_0_) [h]	Volume of treated solution (C_E_ = 0.1C_0_) [L]	Time of sorbent sorption properties depletion (C_E_ = C_0_) [h]	Q (exp) [mg/g]	Q (under static conditions) * [mg/g]
CHs	S1	44	4.4	904	1390.2	2307.0
	S2	46	4.6	924	1504.7	–
CHs-CIT	S1	132	13.2	920	1920.9	2209.0
	S2	82	8.2	906	1339.7	–
CHs-ECH	S1	348	34.8	964	3169.2	2083.0
	S2	346	34.6	968	3212.9	–

*Based on the our previous study^10^.

**Figure 6:**
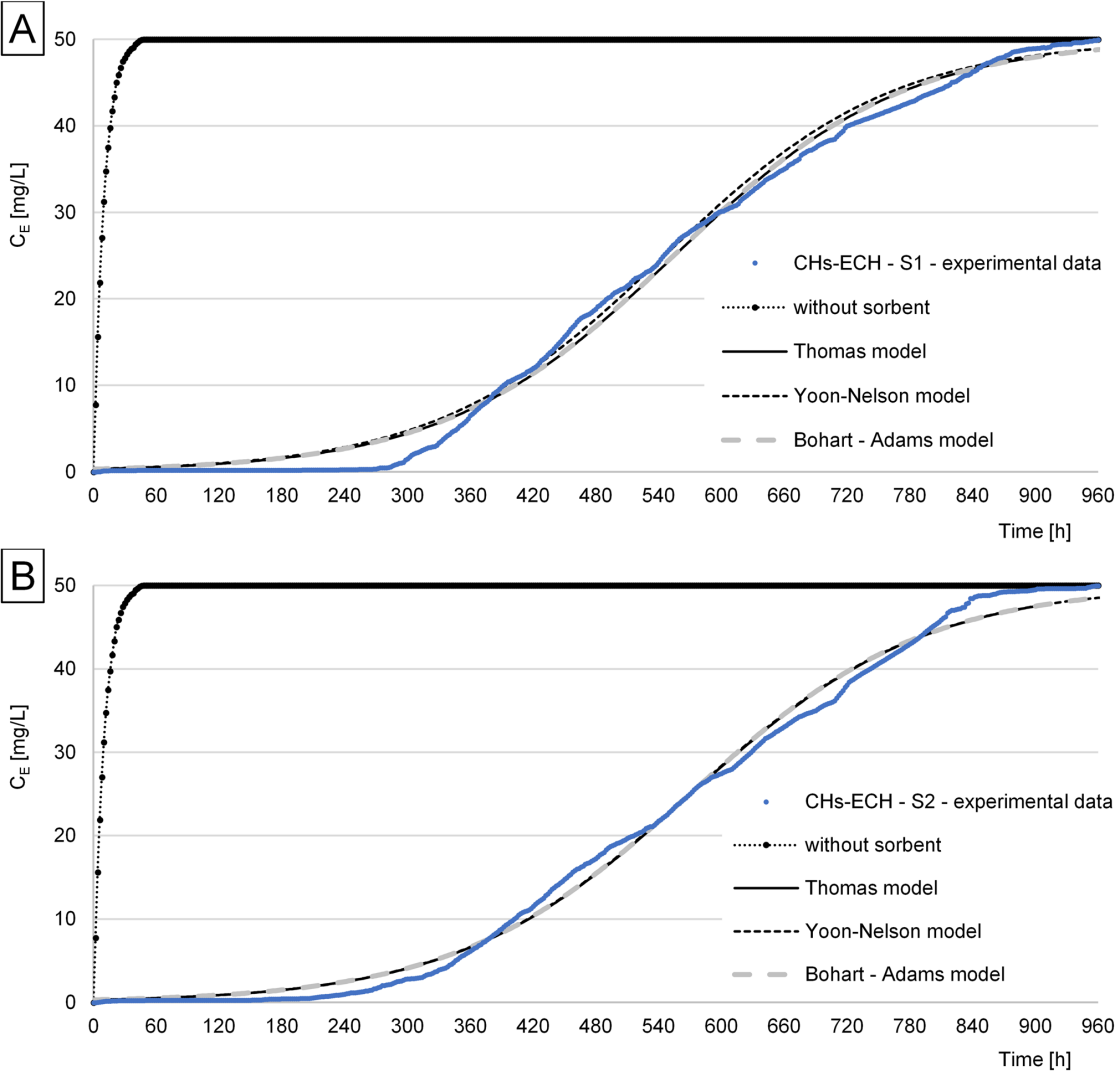
Concentration of RB5 at the outlet from the air-lift with a filling from CHs-ECH: (**A**) S1, (**B**) S2.

Under the flow conditions, the longest period of a high and stable sorption effectiveness was noted in the experimental series with CHs-ECH (Fig. 6). The bed break-through time in the air-lift absorber (effectiveness decrease < 90%) was similar in the case of both solutions tested and reached 348 h for S1 and 346 h for S2. The time after which the CHs-ECH present in the adsorber lost its sorption properties (C_E_ = C_0_) was also alike, reaching 964 h in the experimental series with S1 and 968 h in that with S2 solution (Table 4). The CHs-ECH sorption capacity determined using the Thomson model under flow conditions reached 2777.3 mg RB5/g in S1 and 2855.3 mg RB5/g in S2 (Table 3). Similar results of RB5 sorption from S1 and S2 onto CHs-ECH indicate a negligible effect of S2 composition (NaCl, polyacrylamide) on dye sorption onto the chemically cross-linked chitosan gel. The significantly better sorption properties of CHs-ECH compared to CHs and CHs-CIT are, presumably, due to the pH value of the solutions used in the study (pH 3), which is optimal for the sorption of reactive dyes onto chitosan sorbents^10,13,15,26^.

CHs-ECH exhibited a significantly higher sorption capacity under dynamic (flow) conditions compared to static conditions (Table 4). Under static conditions, the final pH value of sorption fixed at pH 6, which could negatively affect dye binding at the end of the process^15^. The flow conditions used in the study ensured maintaining optimal sorption pH (pH 3.0) throughout the experiment. This, in turn, led to a high effectiveness of RB5 sorption to CHs-ECH over the study period. Ultimately, ECH-CHs ensured a very long period of stable and high RB5 sorption effectiveness and better sorption properties, as clearly noticeable in Fig. 6.

## Summary

The effectiveness of RB5 sorption under flow conditions in the air-lift reactor was largely dependent on sorbent type and sorption pH.

An advantage of using cross-linked chitosan turned out to be the possibility of performing the sorption process at low pH values (pH 3–4), being optimal for RB5 binding. The ionic cross-linking of chitosan using sodium citrate ensured sorbent stability under the flow conditions at pH 4, whereas its covalent cross-linking with epichlorohydrin made the sorbent stable at pH 3. The RB5 sorption onto unmodified chitosan was feasible only at pH > 4 (pH 5).

In the case of the mono-component solutions (S1), the sorption capacities of CHs, CHs-CIT, and CHs-ECH reached 1390.2 mg/g, 1920.9 mg/g, and 3169.2 mg/g, whereas the volume of the solution treated in the adsorber (removal of > 90% RB5) reached 4.4 L, 13.2 L, and 34.8 L, respectively.

The sorption capacity of CHs-ECH obtained under flow conditions (S1) was higher by 52% than that determined under static conditions (Q_max stat_ = 2083 mg/g^10^). This difference could be due to the optimal pH of the solution in the system (pH 3), which—contrary to the static conditions—was maintained throughout the experiment. In turn, the maximal sorption capacities determined under the flow conditions for CHs and CHs-CIT were lower by 40% and 13% than those noted under static conditions (Q_max stat_ = 2307.0 mg/g, Q_max stat_ = 2209.0 mg/g), which was caused by higher sorption pH (pH 5 for CHs and pH 4 for CHs-CIT) used to ensure sorbent stability.

The presence of salt (NaCl, 3 g/L) and the anti-creaser (2 g/L) in the solution (S2) significantly deteriorated RB5 sorption by the ionically cross-linked chitosan (CHs-CIT). The CHs-CIT sorption capacity in S2 was 30% lower than in S1, which was probably due to the interaction of the polyacrylamide contained in the anti-creaser with sodium citrate ions of CHs-CIT. The polyacrylamide accumulated on sorbent’s surface could block dye access to the sorption centers of CHs-CIT. Because of the absence of anionic functional groups in the structure of unmodified chitosan and chitosan cross-linked with epichlorohydrin, the polyacrylamide contained in S2 did not hamper RB5 sorption onto CHs and CHs-ECH.

## Supplementary Information

Supplementary Information.
