# Supplementary material for: The use of air-lift adsorber with a floating filling from a cross-linked chitosan hydrogels for Reactive Black 5 removal
Source: Sci Rep. 2021 Jun 28;11:13382. doi: 10.1038/s41598-021-92856-y (PMC8238981; doi:10.1038/s41598-021-92856-y)
Supplement: Supplementary file 1 — Supplementary Information. [file 41598_2021_92856_MOESM1_ESM.docx]

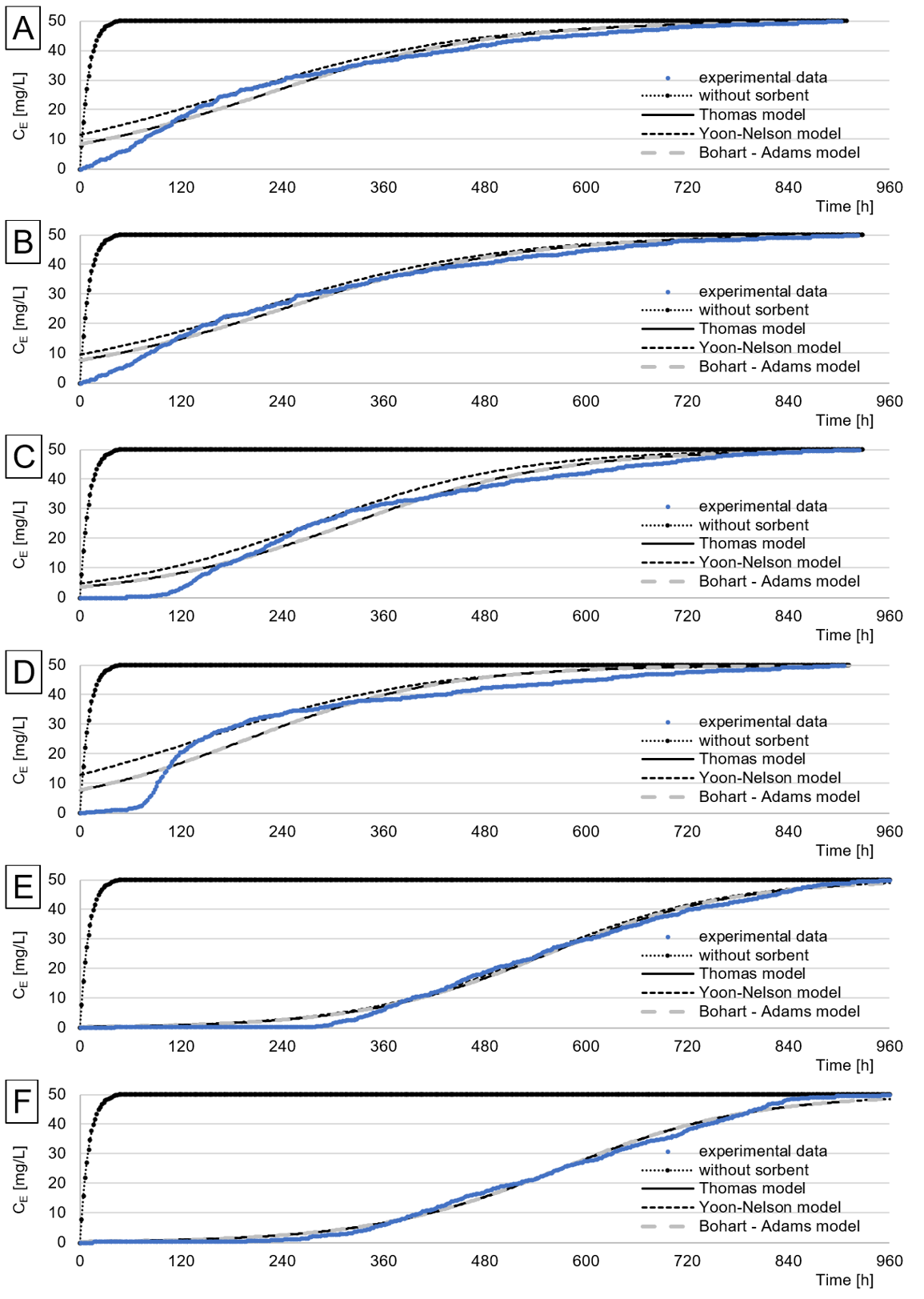


Suppl 1. Concentration of RB5 at the outlet from the air-lift reactor: A) CHs, S1; B) CHs, S2; C) CHs-CIT, S1; D) CHs-CIT, S2; E) CHs-ECH, S1; F) CHs-ECH, S2.
